# Myasthenia Gravis in the Setting of Immune Checkpoint Inhibitor Therapy: Practical Considerations and Opinion-Based Approach to Acute Management

**DOI:** 10.7759/cureus.30638

**Published:** 2022-10-24

**Authors:** Adeel S Zubair, Bhaskar Roy, Joachim M Baehring, Richard J Nowak

**Affiliations:** 1 Neurology, Yale School of Medicine, New Haven, USA

**Keywords:** anti-pdl1, anti-ctla4, anti-pd1, checkpoint inhibitor, myasthenia gravis

## Abstract

Use of immune checkpoint inhibitors (ICI) is increasing in patients with oncologic disease. Three classes of checkpoint inhibitors exist: anti-PD1 (nivolumab, pembrolizumab), anti-CTLA4 (ipilimumab), and anti-PDL1 (atezolizumab, avelumab, durvalumab). ICI therapy has been used in multiple malignancies including renal cell cancer, non-small-cell lung cancer, and melanoma. These therapies have led to improved oncologic treatment and outcomes in patients but can lead to immune-related or inflammatory adverse effects. Neuromuscular system side effects, particularly at the neuromuscular junction, have been observed, including myasthenia gravis (MG).

This narrative review serves to summarize key available information regarding myasthenia gravis in the setting of immune checkpoint inhibitor use including the molecular targets of checkpoint inhibitors, the clinical manifestations of MG in patients with checkpoint inhibitor therapy, and potential treatment options. Studies have shown that the use of checkpoint inhibitor therapy can trigger MG, and that patients with ICI-related MG can have more severe disease.

Recognition and understanding of the range of neurologic complications, including neuromuscular disorders, which can be seen with ICI therapy is a critical step toward developing better treatment algorithms and improved clinical outcomes. Future investigations which include deep mechanistic studies to further our understanding of the immunopathologic triggers and predictive markers of ICI-related MG will be important to address the current knowledge gaps.

## Introduction and background

Immune checkpoints are an integral mechanism employed by cells to prevent development of autoimmunity [1]. These checkpoints are molecules that are used in ensuring immunologic homeostasis [1]. Use of checkpoint inhibitors is increasing in patients with oncologic disease. Starting in 2011, the US Food and Drug Administration (FDA) approved eight immune checkpoint inhibitors in three classes: anti-PD1 (nivolumab, pembrolizumab, cemiplimab, dostarlimab), anti-CTLA4 (ipilimumab), and anti-PDL1 (atezolizumab, avelumab, durvalumab). The pipeline for these medications continues to grow and new therapies are in development. These therapies can be used as monotherapy or in combination. Immune checkpoint inhibitor (ICI) therapy has been used in multiple malignancies including renal cell cancer, non-small-cell lung cancer, and melanoma. These therapies have led to significant anti-malignancy results in patients but can result in immune-related or inflammatory adverse effects against the neuromuscular system [2]. A large case series described 38 patients who had neuromuscular side effects to checkpoint inhibitor therapies [3]. In that series, 5% of cases were fatal with many cases not having full resolution. One such neuromuscular complication is myasthenia gravis [4]. A recent systematic review identified the rate of mortality in patients receiving ICI therapy who developed myasthenic syndromes to be at 28% [5].

Myasthenia gravis (MG) is a T-cell-dependent B-cell-mediated (via IgG autoantibodies) autoimmune disorder which affects the neuromuscular junction. Patients typically present with fluctuating weakness which worsens with fatigue and later in the day [6,7]. MG has a prevalence of approximately 20 per 100,000 in the United States [6-9]. Proteins that are commonly targeted by autoantibodies in the neuromuscular junction (postsynaptic) include the acetylcholine receptor (AChR), muscle-specific kinase (MuSK), and lipoprotein-related protein 4 (LPR4) [6,7].

Herein we review key available information regarding myasthenia gravis in the setting of immune checkpoint inhibitor use including the molecular targets of checkpoint inhibitors, the clinical manifestations of MG triggered by ICI therapy, and potential treatment approaches. Using available data we provide an opinion-based management algorithm.

## Review

Search strategy

References for this narrative review article were identified by searches of PubMed for articles published between 1969 and February 2021. References from relevant articles were also included. The search terms immune checkpoint inhibitor, myasthenia gravis, neuromuscular disorders, and ICI-related MG were used. No language restrictions were used. The final list of references was generated based on relevance to the topics covered in this review.

Molecular targets

There are two important T-cell proteins, CTLA4 and PD-1, which are currently targeted by checkpoint inhibitors; CTLA4 binds to B7-1 or B7-2 whereas PD-1 binds to PD-L1 [2]. This binding allows the tumor cells to evade detection by T-cells, allowing for their unchecked growth [2]. Checkpoint inhibitors inhibit binding of CTLA4 and PD-1 to their receptors via monoclonal antibodies which allow for T-cell recognition and cell death [2]. Currently, the checkpoint inhibitors on the market target PD-1 (pembrolizumab and nivolumab), PD-L1 (atezolizumab, avelumab, and durvalumab), and CTLA4 (ipilimumab).

Checkpoint inhibitor therapy and myasthenia gravis

Studies have shown that the use of checkpoint inhibitor therapy can trigger MG. In patients treated with pembrolizumab, emergence of MG (ocular, generalized, or myasthenic crisis) has been reported to occur after seven to 11 weeks of treatment [10-12]. Use of nivolumab and ipilimumab have also been documented leading to MG [13,14]. While a case report showed evidence of MG in a patient receiving nivolumab and ipilimumab with onset of symptoms at two weeks [15], it remains unclear if combination therapy impacts time of onset or severity of ICI-related MG. Epidemiological studies on ICI-related MG are limited; one large study done at a tertiary US medical center identified that 14 (0.24%) out of a total of 5898 patients treated with ICI developed MG [16].

Patients with pre-existing autoimmune diseases who receive therapy with ICI are susceptible to develop flares of the underlying autoimmunity or new onset immune-related adverse events [16].

ICI-related MG can present with a variety of different conditions; the most common manifestations include ptosis, dyspnea, dysphagia, and limb weakness [16]. ICI-related MG was shown to have a higher Myasthenia Gravis Foundation of America (MGFA) classification (IV/V in 51% of one series) [16]. In idiopathic autoimmune MG, 2-19% of patients reach class IV/V, usually over a period of two to three years [8,17-19].

Treatment with ICI can result in differing rates of adverse events depending on the type of therapy. Monotherapy with PD-1/PD-L1 antibodies induces grade III/IV adverse events in 10-20% of patients whereas monotherapy with anti-CTLA-4 antibodies induces grade III/IV adverse events at a rate of 27% [20-22]. For patients receiving combination immunotherapy, the rate of grade III/IV side effects is higher at 55% [23]. 

While the majority of ICI-related MG cases are anti-acetylcholine receptor (AChR) antibody positive (approximately two-thirds), anti-muscle specific kinase (MuSK) antibody positive and seronegative cases have also been reported [24].

Treatment

Treatment for patients with ICI-related MG is similar to treatment of idiopathic MG [24]. These therapies may include oral corticosteroids, intravenous corticosteroids, intravenous immunoglobulin (IVIg), plasma exchange (PLEX), rituximab, and azathioprine [24-27]. 

**Figure 1:**
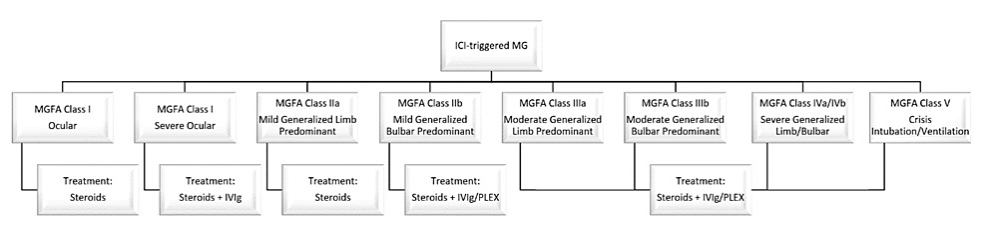
Opinion-based management algorithm for immune checkpoint inhibitor-related myasthenia gravis based on disease status/severity at presentation utilizing the MGFA classification system. MG: myasthenia gravis, MGFA: Myasthenia Gravis Foundation of America, IVIg: intravenous immunoglobulin, PLEX: plasma exchange, ICI: immune checkpoint inhibitor

Currently, the clinical practice guidelines for ICI-related MG recommend addition of IVIg or PLEX if patients do not improve or have worsening with steroids alone, or present with MGFA class III to V [28,29]. Interestingly, one series reported that 95% of patients who received IVIg or PLEX as first-line treatment had clinical improvement versus 65% in those who received steroids alone [16]. That study did show that severity at symptom onset was not predictive of clinical course. Steroid therapy can take weeks to reach maximal clinical benefit and initially may lead to acute worsening of MG symptoms [30,31]. This worsening is transient but can result in progression to respiratory failure in some cases [32]. IVIg and PLEX are used in idiopathic MG and can result in favorable outcomes in patients with severe MG and those with MG exacerbation/crisis [16,33-35]. IVIg/PLEX rescue therapy strategies are typically recommended to mitigate risk of transient worsening especially with high-dose steroids, particularly in patients with moderate-severe disease exacerbation/crisis [30,36,37]. As management decisions in the acute setting can sometimes be challenging, we propose using a standard treatment algorithm based on clinical presentation and symptom severity and as such have tentatively constricted an opinion-based approach (Figure 1).

Corticosteroids given after cessation of ICI therapy do not appear to counteract its immunomodulatory effect although the impact of prolonged or high doses of corticosteroids or use of other immunosuppressants on oncologic prognosis is debated [38,39].

Rechallenge of immune checkpoint inhibitors in patients with MG

Currently, limited information exists in the literature about outcomes in patients with ICI-related MG. A case report documented a 77-year-old man with stage IV melanoma who received pembrolizumab and developed MG after the fourth infusion who was treated with IVIg, pyridostigmine, and prednisone [40]. The pembrolizumab therapy was discontinued with the melanoma in partial response. After tumor relapse and progression, the patient was started on nivolumab along with preventive pyridostigmine and corticosteroid therapy. After 14 doses of nivolumab, the patient had recurrence of MG symptoms which was treated with cessation of nivolumab and increase of corticosteroids [40]. In another series, after symptom resolution, six out of 65 patients were rechallenged with ICI while being maintained on prednisone, pyridostigmine, or IVIg and none of them suffered recurrence of symptoms; however, follow-up period was not reported [16].

Mortality

The mortality reported in the literature regarding ICI-related MG varies. One study of 47 cases found a mortality rate of checkpoint inhibitor-related MG of 29.8% [24]. Other studies have also shown a similar mortality (30.4%) [41]. This is in contrast to idiopathic MG where typical mortality rates are around 6% if patients receive timely treatment [18,42]. Mortality was associated with earlier onset of MG symptoms (typically within the first month of ICI treatment) and with multi-organ toxicity [24].

Myopathies and overlap syndromes in cases of muscle weakness

It is important to recognize that inflammatory myopathies have also been reported in patients receiving immune checkpoint inhibitors [13,43,44]. These patients can present with markedly elevated creatine kinase levels as well as cardiac involvement. Amongst the subtypes of inflammatory myopathies, polymyositis, dermatomyositis, and necrotizing autoimmune myositis are the most common [2]. As patients may also have overlapping MG or MG-like symptoms, we recommend evaluating for both a myopathy and neuromuscular junction disorder [2]. The differential diagnosis of patients presenting with ptosis and diplopia in the setting of ICI therapy should also include orbital myositis. This presentation can often mimic ICI-related MG [45,46]. While this review focuses on myasthenia gravis in the setting of checkpoint inhibitor therapy, it is critically important to also evaluate for possible overlap syndromes.

Myocarditis has also been reported in patients who receive ICI therapy. Patients can present with a range of symptoms from asymptomatic elevations in cardiac biomarkers to life-threatening presentations with cardiogenic shock and arrhythmias [47].

## Conclusions

The incidence of ICI-related MG may continue to increase with wider application of immune checkpoint inhibitors for cancer treatment. Recognition and understanding of the neuromuscular complications associated with these therapies, particularly MG, is a critical step toward developing evidence-based treatment algorithms to improve clinical outcomes. Raising awareness and highlighting current knowledge gaps remain priorities for both the neurology and oncology communities. Further research in this area is needed to inform not only treatment decisions but also to mitigate risk with the use of ICI therapies. 
